# Manipulating Ferroelectric Polarization and Spin Polarization of 2D CuInP_2_S_6_ Crystals for Photocatalytic CO_2_ Reduction

**DOI:** 10.1021/jacs.4c05798

**Published:** 2024-07-25

**Authors:** Chun-Hao Chiang, Cheng-Chieh Lin, Yin-Cheng Lin, Chih-Ying Huang, Cheng-Han Lin, Ying-Jun Chen, Ting-Rong Ko, Heng-Liang Wu, Wen-Yen Tzeng, Sheng-Zhu Ho, Yi-Chun Chen, Ching-Hwa Ho, Cheng-Jie Yang, Zih-Wei Cyue, Chung-Li Dong, Chih-Wei Luo, Chia-Chun Chen, Chun-Wei Chen

**Affiliations:** † Department of Materials Science and Engineering, National Taiwan University, Taipei 10617, Taiwan; ‡ International Graduate Program of Molecular Science and Technology, National Taiwan University (NTU-MST), Taipei 10617, Taiwan; § Molecular Science and Technology Program, Taiwan International Graduate Program (TIGP), Academia Sinica, Taipei 11529, Taiwan; ∥ Department of Chemistry, National Taiwan Normal University, Taipei 11677, Taiwan; ⊥ Center for Condensed Matter Sciences, National Taiwan University, Taipei 10617, Taiwan; # Center of Atomic Initiative for New Materials (AI-MAT), National Taiwan University, Taipei 10617, Taiwan; ∇ Department of Electrophysics, National Yang Ming Chiao Tung University, Hsinchu 300, Taiwan; ○ Department of Electronic Engineering, National Formosa University, Yunlin 632, Taiwan; ◆ Department of Physics, National Cheng Kung University, Tainan 70101, Taiwan; ¶ Graduate Institute of Applied Science and Technology, National Taiwan University of Science and Technology, Taipei 106, Taiwan; †† Department of Physics, Tamkang University, New Taipei City 25137, Taiwan; ‡‡ Institute of Atomic and Molecular Sciences, Academia Sinica, Taipei 10617, Taiwan

## Abstract

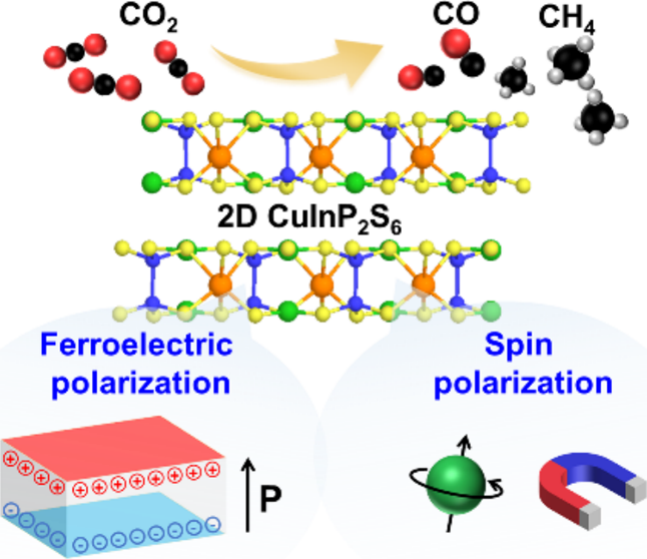

Manipulating electronic polarizations such as ferroelectric or spin polarizations has recently emerged as an effective strategy for enhancing the efficiency of photocatalytic reactions. This study demonstrates the control of electronic polarizations modulated by ferroelectric and magnetic approaches within a two-dimensional (2D) layered crystal of copper indium thiophosphate (CuInP_2_S_6_) to boost the photocatalytic reduction of CO_2_. We investigate the substantial influence of ferroelectric polarization on the photocatalytic CO_2_ reduction efficiency, utilizing the ferroelectric-paraelectric phase transition and polarization alignment through electrical poling. Additionally, we explore enhancing the CO_2_ reduction efficiency by harnessing spin electrons through the synergistic introduction of sulfur vacancies and applying a magnetic field. Several advanced characterization techniques, including piezoresponse force microscopy, ultrafast pump–probe spectroscopy, *in situ* X-ray absorption spectroscopy, and *in situ* diffuse reflectance infrared Fourier transformed spectroscopy, are performed to unveil the underlying mechanism of the enhanced photocatalytic CO_2_ reduction. These findings pave the way for manipulating electronic polarizations regulated through ferroelectric or magnetic modulations in 2D layered materials to advance the efficiency of photocatalytic CO_2_ reduction.

## Introduction

Combating climate change and establishing a sustainable society have become the most crucial challenges for humans to tackle in this century. In particular, the development of renewable energy resources to reduce CO_2_ emissions is essential to achieve this goal. Artificial photosynthesis, which mimics the energy conversion processes of natural plants to use solar-driven CO_2_ reduction to value-added fuels and chemicals, has attracted significant attention recently.^1,2^ However, photocatalytic CO_2_ reduction processes involve sluggish multielectron transfer kinetics and reactions, so the solar-driven CO_2_ reduction conversion efficiency still needs to be improved for practical applications.^3^ The performance of photocatalytic CO_2_ reduction strongly depends on the photogenerated charge separation, transfer, and recombination in the bulk and on the surface of photocatalysts.^4^ Extensive efforts have been devoted to pursuing efficient photocatalysts for CO_2_ reduction by optimizing these key factors among the materials. Constructing heterojunctions with other nanoscale materials to create built-in electric fields at the interfaces of photocatalysts is the commonly used strategy to facilitate charge separation or suppress carrier recombination for enhancing photocatalytic CO_2_ reduction.^5^ Manipulation of electronic polarizations such as ferroelectric polarization or spin polarization within materials to enhance charge separation or reduce carrier recombination is another effective strategy to promote photocatalytic CO_2_ reduction.^6,7^ For example, ferroelectric semiconductors exhibit spontaneous electric polarizations resulting from the displacement of positive and negative charges and have shown promising photocatalytic CO_2_ reduction with an enhanced driving force for charge separation.^8,9^ Recently, our group showed that manipulating electronic spins in magnetic element-doped semiconductors is also an effective strategy to boost photocatalytic CO_2_ reduction efficiencies, resulting from prolonged carrier lifetime and suppressed charge recombination.^10^ Moreover, further manipulation of ferroelectric polarization or spin polarization within photocatalytic materials can be achieved by applying an external electric field or magnetic field (MF), which provides a flexible and controllable strategy to enhance photocatalytic CO_2_ reduction efficiencies.^11^

This work demonstrates a novel approach to achieving efficient photocatalytic CO_2_ reduction using ferroelectric and magnetic modulations on two-dimensional (2D) copper indium thiophosphate (CuInP_2_S_6_, CIPS) crystals. Ferroelectric semiconductors, known for their distinctive spontaneous ferroelectric polarization, have gained significant attention as promising materials for photocatalytic CO_2_ reduction. Most ferroelectric photocatalysts for CO_2_ reduction have been previously reported based on bismuth-based oxide materials like Bi_4_Ti_3_O_12_,^8^ Bi_3_TiNbO_9_,^9^ SrBi_4_Ti_4_O_15_,^12^ and Bi_2_MoO_6_.^13,14^ Recent theoretical simulations have proposed that 2D ferroelectric multilayers, specifically those in 2D van der Waals (vdW) interfaces, exhibit highly efficient photocatalytic CO_2_ conversion due to rapid interlayer charge transfer and separation facilitated by strong interlayer coupling.^15^ The CIPS, a 2D layered material, is particularly intriguing as it retains room-temperature ferroelectric properties even at thicknesses as thin as 4 nm.^16^ Here, by controlling the ferroelectric polarization of 2D CIPS crystals, through either ferroelectric phase transitions or electrical poling, the performance of photocatalytic CO_2_ reduction can be significantly enhanced. In addition to ferroelectric polarization, manipulating unpaired spin electrons of 2D CIPS crystals by introducing sulfur vacancies or applying an external magnetic field also enhances the photocatalytic CO_2_ reduction performance. The advanced characterization techniques, including piezoresponse force microscopy, ultrafast pump–probe spectroscopy, *in situ* X-ray absorption spectroscopy (XAS), and *in situ* diffuse reflectance infrared Fourier transformed (DRIFT) spectroscopy, were performed to unveil the underlying mechanism on the enhanced photocatalytic CO_2_ reduction. Our result suggests that manipulation of ferroelectric polarizations and spin polarizations on 2D CIPS photocatalysts may effectively boost CO_2_ photoreduction efficiencies.

## Results and Discussion

**Figure 1:**
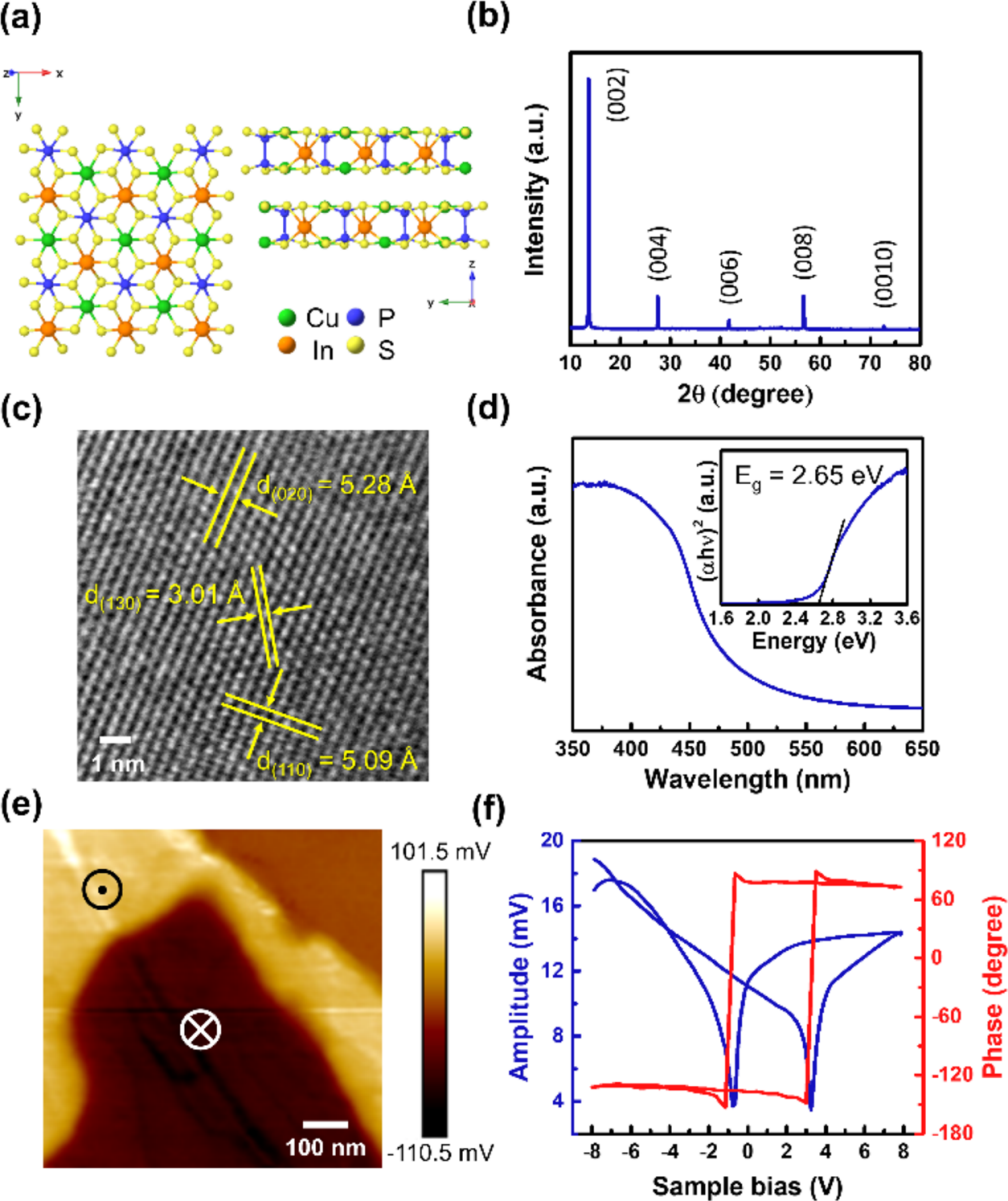
Characterizations of the 2D CIPS crystal. (a) Schematic of atomic structure from top and side views of CIPS. Green, orange, blue, and yellow atoms represent Cu, In, P, and S atoms, respectively. (b) XRD pattern of single-crystal CIPS. (c) High-resolution TEM image of exfoliated CIPS flake. The *d*-spacing of (110), (020), and (130) planes are labeled. (d) Absorbance spectrum measured by UV–vis spectrometer and the corresponding Tauc plot for extracting band gap. (e) OP PFM image of exfoliated CIPS flake with a thickness of 200 nm. (f) Local PFM amplitude and phase hysteresis loops.

CIPS is a type of metal thiophosphate that belongs to the family of 2D vdW layered structures. The schematic of the CIPS atomic structure is shown in Figure 1a. The atomic structure constitutes the [P_2_S_6_]^4–^ anion sublattice, along with Cu^+^ and In^3+^ cation sublattices. Within the anion sublattice, the sulfur atoms form the octahedral voids, in which Cu^+^ and In^3+^ fill the two-thirds and P–P triangular pairs serve the other one-third.^17,18^ The CIPS single crystal used in this work was synthesized through the chemical vapor transport (CVT) method.^19^ Additional details on the CVT growth are given in the Supporting Information. The layered nature of CIPS is evident in the single-crystal X-ray diffraction (XRD) pattern, which exclusively shows Bragg’s reflection of (00*l*) planes (Figure 1b). For transmission electron microscopy (TEM) analysis, a bulk crystal is mechanically exfoliated into microflakes on a copper grid (Figure S1a). The high-resolution TEM image in Figure 1c reveals the *d*-spacing of 3.01, 5.09, and 5.28 Å for (130), (110), and (020) planes, respectively.^20,21^ The selected area electron diffraction (SAED) pattern, as shown in Figure S1b, exhibits a dotted pattern resembling the previously simulated electron diffraction pattern,^21^ representing the crystalline structure of thin CIPS. Additionally, Figure S2 displays the Raman spectrum of the CIPS crystal consisting of various phonon modes.^19,22^Figure 1d exhibits the ultraviolet–visible (UV–vis) absorption spectrum of CIPS, and the optical band gap determined from the Tauc plot is 2.65 eV (inset of Figure 1d). Besides, the CIPS is recognized as a room-temperature ferroelectric material with spontaneous polarization, particularly in the out-of-plane (OP) direction.^16^ The spontaneous OP polarization originates from the off-centering displacement of the copper ions, breaking the lattice inversion symmetry within the individual layer.^17^ The ferroelectricity of 2D CIPS is measured using piezoresponse force microscopy (PFM) in contact-resonance mode. Figure 1e displays the OP PFM image, illustrating the piezoresponse of CIPS in the OP direction. The bright and dark contrast domains in this image correspond to the upward (+mV) and downward (−mV) OP polarizations, respectively. The weak net piezoresponse in the upper right corner of the image may be attributed to the presence of antiferroelectric ordering.^23^ The corresponding topography, PFM amplitude (piezoresponse strength), and PFM phase (piezoresponse polarity) images can be found in Figure S3. Typically, ferroelectric polarization is switchable, manifested by the corresponding ferroelectricity. Figure 1f shows the switchable ferroelectricity within 2D CIPS, as evidenced by the distinctive butterfly-shaped amplitude loop and the phase reversal observed at coercive voltages. The material characterizations described above suggest that 2D CIPS crystals exhibit unique physical properties, including spontaneous ferroelectric polarization at room temperature and an appropriate band gap for light harvesting, making them a promising candidate for photocatalytic applications. Moreover, the curves measured by contact Kelvin probe force microscopy (cKPFM) in Figure S4 with the remnant offset at zero voltage and a nonlinear hysteresis loop eliminate the possibility of fake hysteresis signals caused by electrostatic charge injection effects and further confirm the ferroelectricity of CIPS.^24^

**Figure 2:**
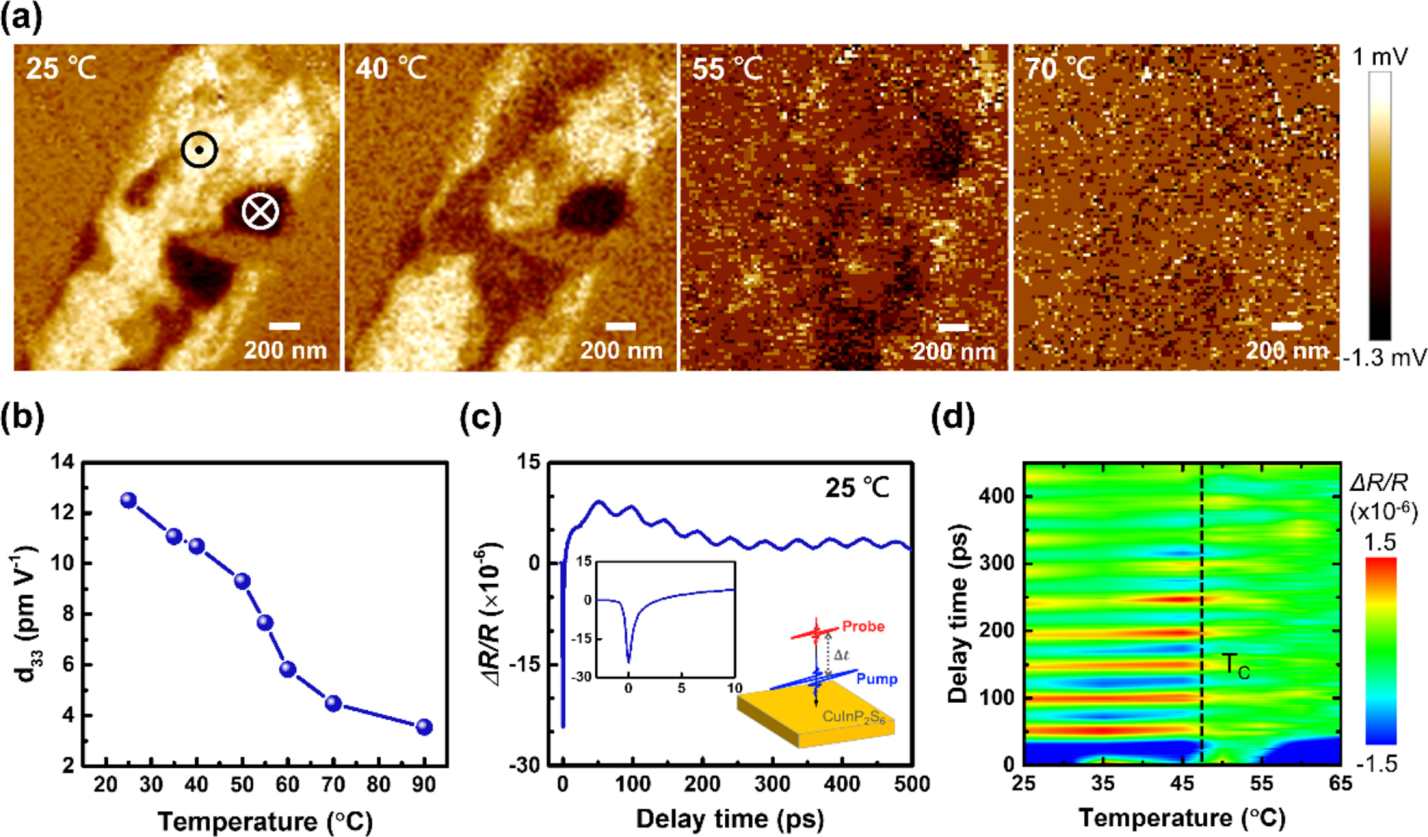
Ferroelectricity of the CIPS. (a) OP PFM images at 25, 40, 55, and 70 °C. (b) Temperature-dependent piezoelectric coefficient *d*_33_. (c) Transient reflectivity changes (Δ*R*/*R*) of CIPS at room temperature. (d) Transient reflectivity changes (Δ*R*/*R*) as a function of delay time at temperatures from 25 to 65 °C.

Typically, the spontaneous polarization of ferroelectric materials can be controlled or manipulated through various means, including temperature, pressure, or the application of an external electric field.^25^ The CIPS may undergo a first-order phase transition from an ordered ferroelectric to disordered paraelectric phases when the temperature exceeds Curie temperature (*T*_C_), which was reported to be ∼42 °C.^16,26−28^ The ferroelectric-paraelectric phase transition of 2D CIPS can also be observed when we conduct temperature-dependent PFM measurements. Figure 2a illustrates the OP PFM images of a CIPS flake measured at 25, 40, 55, and 70 °C. At room temperature 25 °C, the polarization domains show a clear contrast. A similar PFM image is also observed at 40 °C. However, as the temperature rises to 55 °C, the piezoresponse significantly reduces and nearly disappears at 70 °C due to the phase transition. To quantitatively evaluate the corresponding piezoelectric coefficient, *d*_33_, at varied temperatures, as shown in Figure 2b, the PFM signals were taken in off-resonance modes. The *d*_33_ value decreases from 12.5 pm V^–1^ at 25 °C to 3.5 pm V^–1^ at 90 °C, which is consistent with the contrast change in Figure 2a, as a result of the ferroelectric-paraelectric phase transition of CIPS across *T*_C_.

Moreover, we employed ultrafast pump–probe spectroscopy to examine the creation of an internal electric field from the ferroelectric polarization of 2D CIPS. Figure 2c shows the transient reflectivity change (Δ*R*/*R*) signal in the time domain. The measured Δ*R*/*R* signal can be seen as a summation of an exponential decay and a damping oscillation.^29^ This long-living oscillation originates from the spatial separation of photogenerated electrons and holes by an internal electric field.^30,31^ The electron and hole distribution change perturbs the lattice configuration equilibrium, induces mechanical stress, and generates coherent acoustic phonons, which cause the oscillatory modification of the dielectric properties and a change in reflectivity in the time domain.^30,31^ The observed oscillation in the Δ*R*/*R* signal is a distinctive signature of ferroelectric polarization. Figure 2d presents a 2D contour plot of the Δ*R*/*R* signals as a function of the temperature. The oscillations observed in the Δ*R*/*R* signals gradually weaken and nearly vanish as the temperature exceeds the transition temperature *T*_C_, consistent with the ferroelectric-paraelectric phase transition obtained from temperature-dependent PFM measurements.

**Figure 3:**
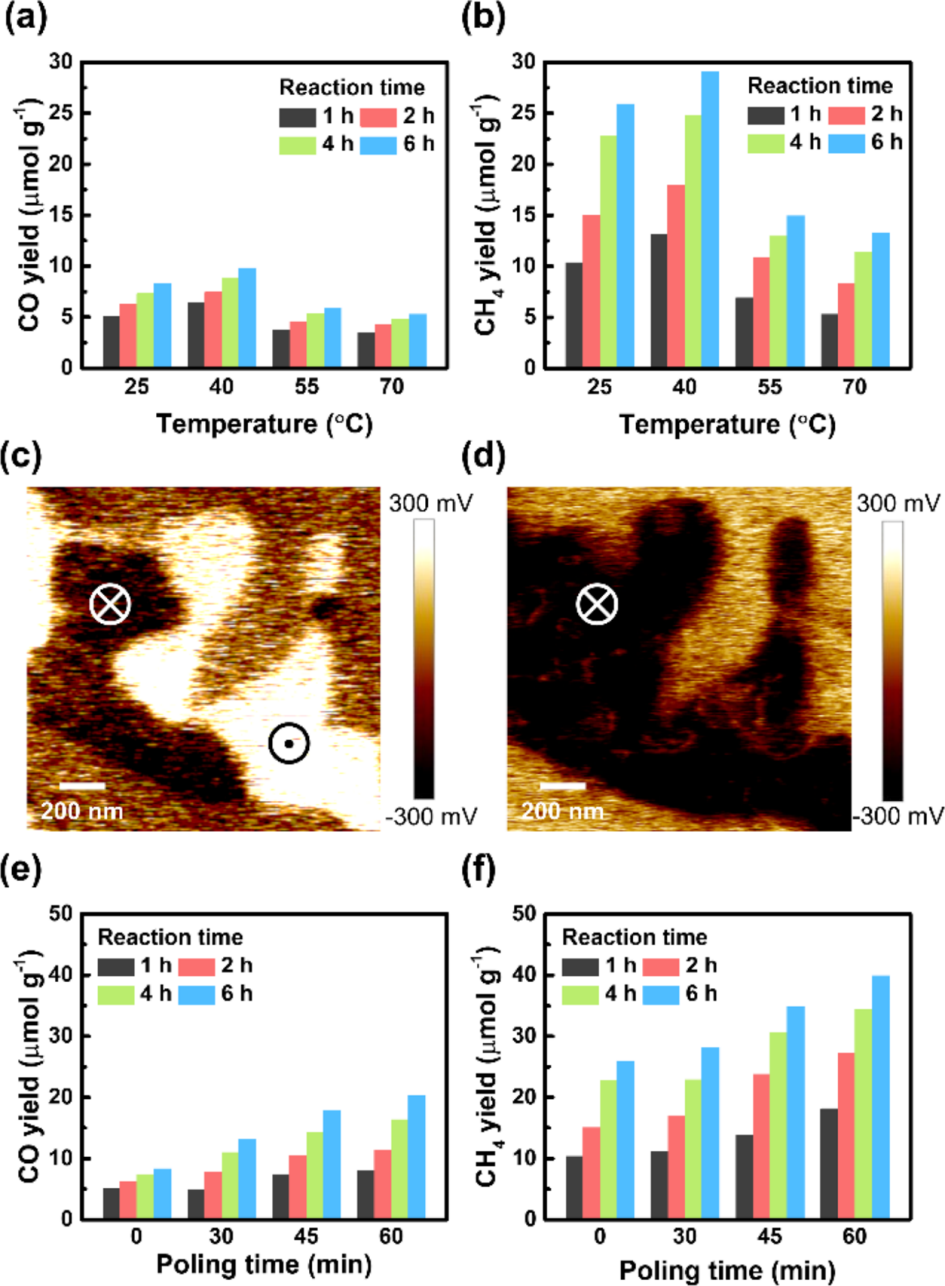
Photocatalytic CO_2_ reduction performance of ferroelectric CIPS. (a) CO yield and (b) CH_4_ yield at 25, 40, 55, and 70 °C after 1, 2, 4, and 6 h reactions. The OP PFM images (c) before and (d) after electrical poling. (e) CO yield and (f) CH_4_ yield with poling times of 0, 30, 45, and 60 min.

Polarization is a critical factor in driving the performance of ferroelectric photocatalysts, particularly in reactions such as water splitting^32,33^ and CO_2_ reduction.^8,9,12−14^ The polarization efficiently separates photogenerated electron–hole pairs, suppresses carrier recombination, prolongs carrier lifetimes, and significantly enhances the conversion efficiency of solar energy into chemical energy.^6,34^ The photocatalytic CO_2_ reduction (gas/solid reaction) experiments of 2D CIPS ferroelectric crystals were performed in a closed chamber with 30 min prepurging CO_2_/H_2_O mixture gas. Before each experiment, the chamber was purged for 30 min with a mixture of CO_2_ and H_2_O gases. Each sample was subjected to separate reaction batches lasting for 1, 2, 4, and 6 h. The products during these reactions were collected and subsequently analyzed using a gas chromatography–mass spectrophotometer (GC-MS). The corresponding calibration curves for the CO and CH_4_ gases were established beforehand, as presented in Figure S5. The results of our product analysis reveal that CIPS can convert CO_2_ into CO and CH_4_ gases. No CO and CH_4_ products were detected from the control batches without light illumination and purging with N_2_ gas. In addition, the isotopic mass spectra of reaction products using ^13^CO_2_ as the reactant also support the CO_2_ reduction originating from CIPS (Figure S6). Figure 3a,b exhibits the CO and CH_4_ product yields of CIPS under photocatalytic CO_2_ reduction measurements at different temperatures. At 25 °C, the CIPS shows the yield rates of 5.05 μmol g^–1^ h^–1^ for CO and 10.38 μmol g^–1^ h^–1^ for CH_4_ within the first hour and achieves the product yields of 8.31 μmol g^–1^ for CO and 25.9 μmol g^–1^ for CH_4_ after 6 h of reaction time. As the temperature increases to 40 °C, the yields of CO and CH_4_ reach 9.82 and 29.04 μmol g^–1^, respectively, after 6 h of reaction time. The enhanced product yields can be attributed to the thermodynamic effect, resulting from the higher probability of reactants overcoming the activation energy barriers.^35^ However, the photocatalytic performance drops significantly as the temperature is further raised to 55 and 70 °C. This observation can be correlated to the ferroelectric-paraelectric phase transition. Despite thermal enhancement at higher temperatures, the paraelectric phase CIPS (at 55 and 70 °C) exhibits lower product yields for CO_2_ reduction compared to the ferroelectric phase CIPS counterparts (at 25 and 40 °C). The result indicates the importance of ferroelectric polarization on the photocatalytic CO_2_ reduction performance. Consequently, the 2D CIPS demonstrates superior photocatalytic activity for CO_2_ reduction below the transition temperature due to more efficient charge separation processes resulting from ferroelectric polarization.

In addition to the phase transition, the ferroelectric domain is another important factor affecting the polarization of materials. Ferroelectric domains correspond to small regions within the ferroelectric material, where the electric dipoles are uniformly oriented. These domains may vary in size and shape. Within each domain, the electric dipoles point in a specific direction, creating a net electric polarization. Figure 3c exhibits the OP PFM image of the CIPS, where bright and dark domains represent the upward and downward polarization directions. Although polarization in ferroelectric materials can create an internal electric field to promote charge separation of photogenerated electrons and holes, the domain boundaries where the adjacent ferroelectric domains have different dipole orientations may act as sites for electron–hole recombination. Accordingly, the overall charge separation efficiency is reduced. Corona poling is a technique used to align these domains in a controlled manner by applying a high-voltage corona discharge to the surface of ferroelectric material. Here, we employed the corona poling process on the CIPS by applying a strong electric field to align the OP ferroelectric polarization direction. Figure 3c exhibits the PFM image of the CIPS sample before corona poling, where both upward and downward polarizations can be seen. By contrast, Figure 3d shows that the OP domain becomes dark after corona poling, indicating the successful establishment of the OP ferroelectric domains with a preferred downward polarization direction. The photocatalytic CO_2_ reduction performances of 2D CIPS were further evaluated by varying the poling time of 30, 45, and 60 min. The corresponding yields of CO and CH_4_ are shown in Figure 3e,f. Both the CO and CH_4_ yields increase with increased poling time. For a poling time of 60 min, the highest CO and CH_4_ yields are reached with 20.34 and 39.88 μmol g^–1^, respectively, for a 6 h reaction. The improved photocatalytic CO_2_ reduction efficiencies observed after electrical poling primarily arise from aligning the polarization direction of ferroelectric domains with a more favorable orientation. When the poling duration exceeds 60 min, a decline in the CO_2_ reduction performance was observed, attributed to overexposure to the high-voltage discharge. The aforementioned findings suggest that manipulating the ferroelectric polarization of 2D CIPS crystals, through either the control of temperature or the application of an external electric field, is an effective method for enhancing photocatalytic CO_2_ reduction.

**Figure 4:**
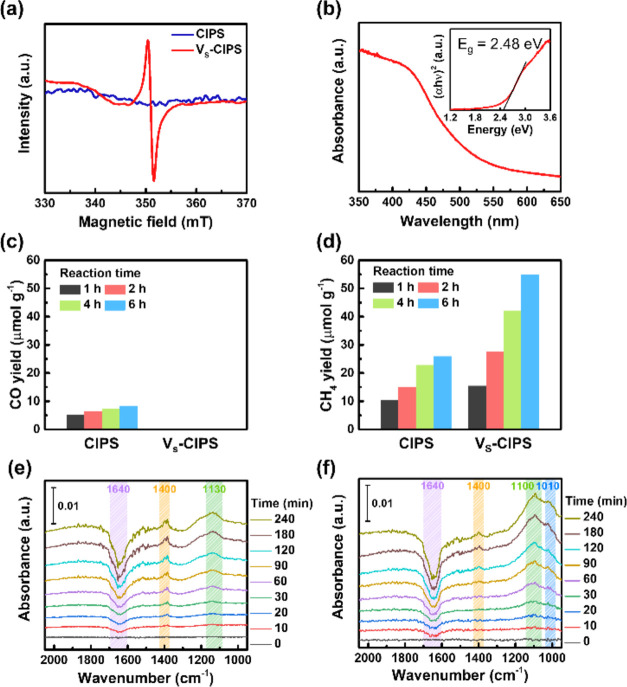
V_S_-CIPS and its photocatalytic CO_2_ reduction performance compared with pristine CIPS. (a) EPR spectra of CIPS and V_S_-CIPS. (b) Absorbance spectrum and the corresponding Tauc plot of the V_S_-CIPS. (c) CO yield and (d) CH_4_ yield of CIPS and V_S_-CIPS at room temperature after 1, 2, 4, and 6 h reactions. The *in situ* DRIFT spectra of the (e) pristine CIPS and (f) V_S_-CIPS with light irradiation time.

Spin polarization, by manipulating the spin states of electrons in materials, has been recently reported as another degree of electronic freedom to improve the performances of electrocatalysts and photocatalysts.^10,36−39^ In photocatalysis, the manipulation of electronic spin can influence charge separation and reduce the recombination of photogenerated carriers when photocatalysts absorb light. Introducing specific dopants or modifying the surface of the photocatalysts with spin-active species can promote spin polarization. Doping with transition metal ions is a common approach to achieve this goal.^10,40^ Also, vacancy (or defect) engineering is another strategy to increase unpaired spin electrons and enhance photocatalytic CO_2_ reduction. Localized electrons in defect states provide sufficient catalytic activity for the adsorption of water and CO_2_ molecules. These defects, which consist of unpaired spin electrons, can be further manipulated through magnetic field modulation.^10,36^ Here, we introduce sulfur vacancy in CIPS through an annealing process in an inert atmosphere due to the low formation energy of sulfur vacancy in CIPS even than MoS_2_.^41^ The CIPS with sulfur vacancies (denoted as V_S_-CIPS) exhibits no substantial alteration in its crystal structure, as confirmed by XRD analysis (Figure S7). However, the Raman peaks in Figure S8 become weaker and broader when compared to the pristine CIPS. The electron paramagnetic resonance (EPR) spectrum in Figure 4a for V_S_-CIPS displays a signal at approximately 350 mT (with a *g*-factor of 2.003), indicating the presence of unpaired electrons trapped near sulfur vacancies.^42,43^ By contrast, no EPR signal is detected in the pristine CIPS before the annealing process. The concentration of sulfur vacancies was determined to be approximately 12 mol % via inductively coupled plasma mass spectrometry (ICP-MS) by comparing the element ratios with pristine CIPS. The optical band gap determined from the absorbance spectrum in Figure 4b is 2.48 eV, slightly smaller than that of pristine CIPS (2.65 eV) owing to the localized defect states induced near the top of the valence band.^41^

We performed photocatalytic CO_2_ reduction on the pristine CIPS and the V_S_-CIPS. Figure 4c,d presents their corresponding product yields. Interestingly, the introduction of sulfur vacancies on CIPS not only enhances the product yields but also alters the selectivity of photocatalytic CO_2_ reduction. During photocatalytic CO_2_ reduction, only the CH_4_ gas, without any other reduction byproducts, was detected for V_S_-CIPS. In contrast, both the CO and CH_4_ products were collected for the pristine CIPS under the same experimental conditions. V_S_-CIPS exhibited a nearly 100% selectivity toward CH_4_ for V_S_-CIPS with a product yield of 54.9 μmol g^–1^ after a 6 h reaction, which is approximately twice the CH_4_ product yield of the pristine CIPS. Recently, Li et al. reported on a sulfur-deficient bimetal catalyst CuIn_5_S_8_, which features charge-enriched Cu–In dual sites.^44^ This catalyst exhibits high selectivity for the photocatalytic production of CH_4_ from CO_2_, arising from the formation of a highly stable Cu–C–O–In intermediate at the sulfur-deficient Cu–In dual sites.^44^ Because our V_S_-CIPS consists of similar dual metals of Cu and In, the high selectivity of CH_4_ production for V_S_-CIPS compared to that of the pristine CIPS in the photocatalytic CO_2_ reduction can also be attributed to the presence of sulfur-deficient Cu–In dual sites due to sulfur vacancies. Figure 4e,f shows the *in situ* DRIFT spectra of the CIPS and V_S_-CIPS, which were utilized to monitor the reaction intermediates.^45,46^ The DRIFT spectra of the CIPS and V_S_-CIPS were acquired with light irradiation time after purging the CO_2_/H_2_O gas mixture (Figure 4e,f). Upon light irradiation, the intensity of a peak at around 1640 cm^–1^ dramatically decreases. This decreasing peak could be associated with surface-adsorbed water (HOH bonding) and chemisorbed CO_2_ species,^45,47^ which implies the initial stage of the CO_2_ reduction process. Various surface-adsorbed species, including *COOH at 1400 cm^–1^,^48−52^ *CH_3_O at 1130 and 1100 cm^–1^,^44,53−56^ and *CHO at 1010 cm^–1^ are formed during photocatalytic CO_2_ reduction reaction.^51,57,58^ All of these species are the key intermediates for converting CO_2_ to CH_4_.^51,53,55^ The intensity of each peak gradually increases with the extension of the light irradiation time. The more intense peaks on the V_S_-CIPS suggest a higher yield of CH_4_ production than that of the pristine CIPS. Additionally, V_S_-CIPS exhibits a superior solar-to-fuel efficiency in the CO_2_ reduction reactions. Based on the product yields after a 6 h reaction time, V_S_-CIPS utilizes almost twice the number of electrons in the CO_2_ reaction than the pristine CIPS (439.2 μmol g^–1^ for V_S_-CIPS and 223.8 μmol g^–1^ for pristine CIPS). The above result suggests that defect engineering by creating sulfur vacancies in CIPS significantly enhances the photocatalytic CO_2_ reduction performance.

**Figure 5:**
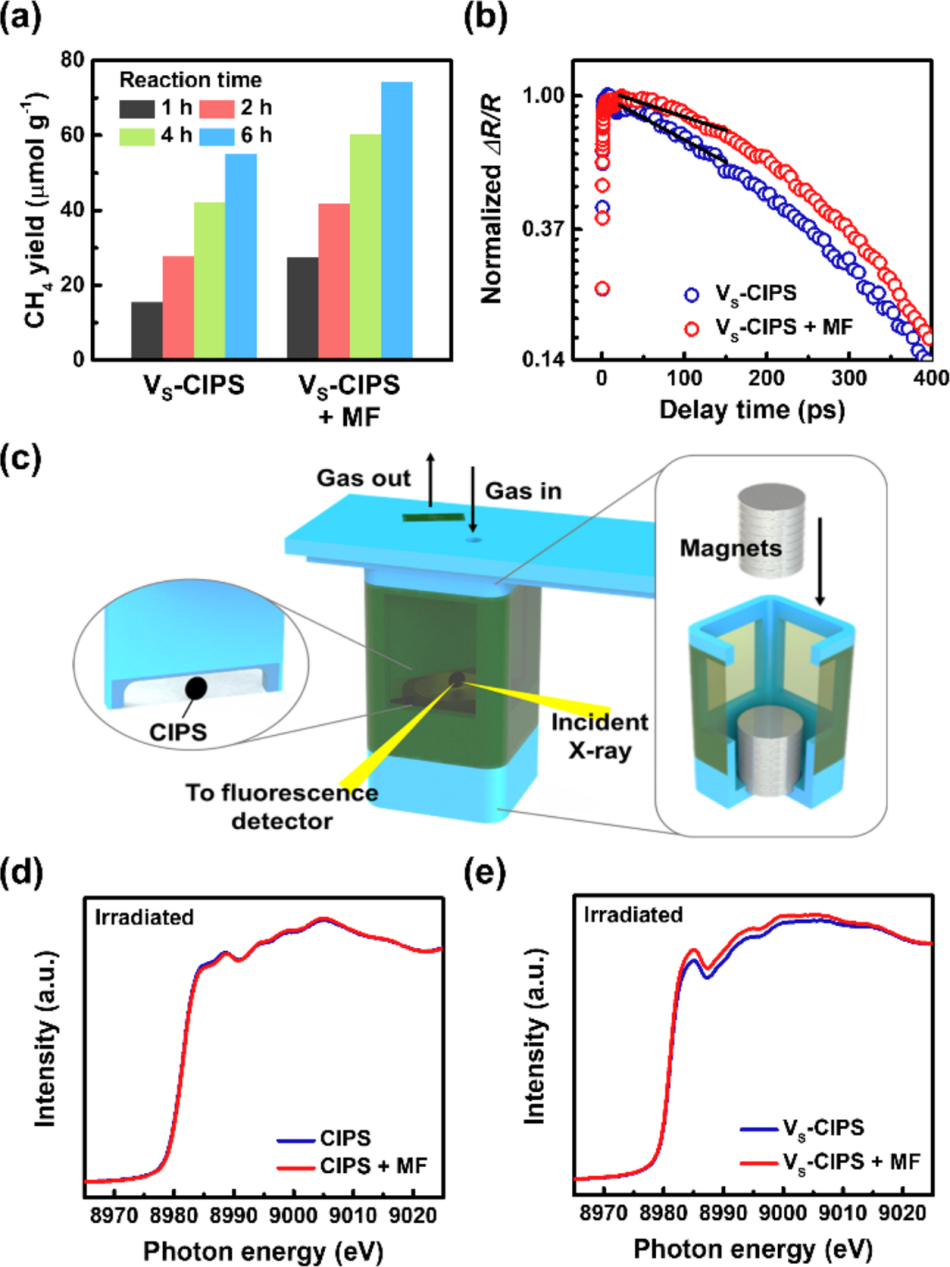
Magnetic field-enhanced CO_2_ reduction performance. (a) CH_4_ yield of the V_S_-CIPS and V_S_-CIPS with a magnetic field after 1, 2, 4, and 6 h reactions. (b) Normalized transient reflectivity changes (Δ*R*/*R*) of the V_S_-CIPS without and with a magnetic field. (c) Schematic illustration of the *in situ* XAS measurement. *In situ* irradiated XAS at the Cu K-edge of (d) pristine CIPS and (e) V_S_-CIPS without and with a magnetic field. The applied magnetic field is 300 mT.

The advantages of defect engineering in CIPS can be summarized as follows: (i) reducing the band gap of V_S_-CIPS to increase light-harvesting efficiency, (ii) enriching the density of surface-active centers for the adsorption of water and CO_2_ molecules to facilitate CO_2_ reduction, and (iii) increasing the number of unpaired spin electrons to enhance spin polarization. To discern the influence of spin polarization on enhanced photocatalytic CO_2_ reduction among the above three factors, we further investigate the photocatalytic performance of V_S_-CIPS under an external magnetic field. The V_S_-CIPS exhibits an increased product yield of CH_4_ gas from 54.9 μmol g^–1^ (without a magnetic field) to 74.29 μmol g^–1^ (by applying the permanent magnets with a magnetic field of 300 mT) after a 6 h reaction (Figure 5a). The result indicates that the photocatalytic CO_2_ reduction performance of V_S_-CIPS can be further enhanced by applying an external magnetic field. By contrast, no such correlation is found over the pristine CIPS (Figure S9). The ultrafast pump–probe measurement verified the corresponding carrier dynamics of V_S_-CIPS with and without an external magnetic field. As shown in Figure 5b, the photoinduced Δ*R*/*R* signals reveal that the photoexcited electrons in the conduction band release their energy through the intraband electron–electron scattering, and the excitons decay via the phonon-assisted nonradiative recombination.^59^ As a result, a prolonged nonradiative recombination lifetime can be observed for V_S_-CIPS under a magnetic field of 300 mT, compared to V_S_-CIPS without a magnetic field. The increased spin polarization of electrons in V_S_-CIPS induced by an external magnetic field prolongs the lifetime of photogenerated charge carriers, leading to a substantial reduction in carrier recombination. Consequently, the prolonged carrier lifetime may promote the diffusion of photogenerated carriers to the surface, facilitating crucial redox reactions for photocatalytic CO_2_ reduction.

The enhanced photocatalytic CO_2_ reduction performance of V_S_-CIPS under a magnetic field is further investigated by *in situ* XAS measured using synchrotron radiation (BL01C at Taiwan Light Source (TLS) of National Synchrotron Radiation Research Center (NSRRC), Taiwan). Here, the *in situ* XAS on the pristine CIPS and V_S_-CIPS samples under light irradiation with and without magnetic field was performed to understand the effect of magnetic field modulation on improving photocatalytic CO_2_ reduction (Figure 5c). Figure 5d,e exhibits the *in situ* irradiated XAS at the Cu K-edge of pristine CIPS and V_S_-CIPS, respectively. Comparing the absorption edge energy in both spectra with various Cu-oxides references (Figure S10) reveals that the Cu(I) state with a filled 3d^10^ electronic configuration predominantly exists in both pristine CIPS and V_S_-CIPS. A closer examination of the rising absorption edge suggests that the energy of V_S_-CIPS is lower than that of the pristine CIPS, indicating the presence of sulfur vacancies in V_S_-CIPS.^60^ The formation of sulfur vacancies may cause an increased number of unpaired spin electrons.^61,62^ The red curves in Figure 5d,e correspond to the *in situ* irradiated XAS under an external magnetic field (300 mT). It is found that the magnetic field has a negligible effect on the photogenerated charge carriers of the pristine CIPS because the spectra with and without applied magnetic fields are nearly identical. On the contrary, the spectral intensity of V_S_-CIPS is enhanced under an external magnetic field, which could be attributed to more efficient charge carriers transfer to the essential intermediates CO_2_ species. The presence of unpaired spin electrons in V_S_-CIPS can be further modulated by applying an external magnetic field to enhance photocatalytic CO_2_ reduction, while this effect is nearly negligible for the pristine CIPS without a sulfur vacancy. The result is consistent with the above observation, with the prolonged carrier lifetime and reduced carrier recombination of V_S_-CIPS under an external magnetic field due to increased spin polarization. Accordingly, the transfer of photogenerated electrons and holes to the redox reactants becomes more effective, thereby facilitating the CO_2_ reduction reaction.

Nevertheless, it is worth noting that the creation of defects in CIPS may also inevitably cause lattice distortion and simultaneously reduce spontaneous ferroelectric polarization. This can be evident from the absence of oscillations in the pump–probe Δ*R*/*R* signals as seen in Figure 5b (V_S_-CIPS) as compared to that in Figure 2c (pristine CIPS). Here, we attempted to restore the ferroelectric polarization of V_S_-CIPS by post-treatment with electrical poling. While achieving complete recovery of the ferroelectric polarization in V_S_-CIPS may not be feasible, it is still possible to enhance the ferroelectric polarization of V_S_-CIPS through postpoling treatment. Figure S11a shows the resulting OP PFM image of the V_S_-CIPS after the postpoling treatment, where the appearance of the dark OP domains with relatively strong piezoresponse amplitude indicates the restoration of ferroelectric polarization after treatments. Finally, through the synergistic manipulation of ferroelectric polarization and spin polarization, the V_S_-CIPS sample subjected to postpoling treatment and placed under a magnetic field demonstrates an improvement in photocatalytic CO_2_ reduction performance, showing a CH_4_ yield of 82.39 μmol g^–1^ after a 6 h reaction (denoted as V_S_-CIPS + poling + MF in Figure S11b). The result suggests that regulating ferroelectric or spin polarization by applying an electric or magnetic field provides a flexible and controllable strategy to enhance photocatalytic CO_2_ reduction efficiencies.

## Conclusions

In summary, we have demonstrated the capability to manipulate both ferroelectric and spin polarizations in 2D CIPS, effectively enhancing the photocatalytic reduction of CO_2_. By controlling the ferroelectric-paraelectric phase transition and applying electrical poling, we observed a substantial influence of ferroelectric polarization on solar-to-fuel efficiency. Additionally, with the synergistic introduction of sulfur vacancies and application of a magnetic field, the photocatalytic CO_2_ reduction efficiencies of V_S_-CIPS are largely improved compared to those of the pristine counterpart due to increased spin polarization. Our findings pave the way for manipulating electronic polarizations by ferroelectric and magnetic field modulations in 2D layered materials, offering promising avenues for improving the efficiency of photocatalytic CO_2_ reduction.

## Supporting Information Available

Experimental methods, TEM image and SAED pattern, Raman spectrum, piezoresponse amplitude and phase images of pristine CIPS, cKPFM curves, calibration curves for CO and CH_4_ gas concentrations, isotropic mass spectra of ^13^CO and ^13^CH_4_, XRD pattern of V_S_-CIPS, CO and CH_4_ yields of pristine CIPS without and with a magnetic field, XAS Cu K-edge spectra with Cu-oxides references, and PFM image and CH_4_ yield of V_S_-CIPS after postpoling (PDF)
