# Supplementary material for: Manipulating Ferroelectric Polarization and Spin Polarization of 2D CuInP2S6 Crystals for Photocatalytic CO2 Reduction
Source: J Am Chem Soc. 2024 Jul 25;146(33):23278–88. doi: 10.1021/jacs.4c05798 (PMC11345765; doi:10.1021/jacs.4c05798)
Supplement: Supplementary file 1 — ja4c05798_si_001.pdf [file ja4c05798_si_001.pdf]

Supporting Information

Manipulating Ferroelectric Polarization and Spin Polarization of 2D $CuInP_2S_6$ Crystals for Photocatalytic CO_2 Reduction

Chun-Hao Chiang,[†,‡] Cheng-Chieh Lin,[#,±,‡] Yin-Cheng Lin,[†] Chih-Ying Huang,[#,±] Cheng-Han Lin,[∥] Ying-Jun Chen,[∥] Ting-Rong Ko,[¶] Heng-Liang Wu,[#,¶,§] Wen-Yen Tzeng,[▲,◊] Sheng-Zhu Ho,[□] Yi-Chun Chen,[□,*] Ching-Hwa Ho,[&,*] Cheng-Jie Yang,[∆] Zih-Wei Cyue,[†] Chung-Li Dong,[∆] Chih-Wei Luo,[▲] Chia-Chun Chen,[∥,♦,*] and Chun-Wei Chen[†,#,¶,§,*]

† Department of Materials Science and Engineering, National Taiwan University, Taipei 10617, Taiwan

International Graduate Program of Molecular Science and Technology, National Taiwan University (NTU-MST), Taipei 10617, Taiwan

± Molecular Science and Technology Program, Taiwan International Graduate Program (TIGP), Academia Sinica, Taipei 11529, Taiwan

∥ Department of Chemistry, National Taiwan Normal University, Taipei 11677, Taiwan

¶ Center for Condensed Matter Sciences, National Taiwan University, Taipei, 10617, Taiwan

§ Center of Atomic Initiative for New Materials (AI-MAT), National Taiwan University, Taipei, 10617, Taiwan

▲ Department of Electrophysics, National Yang Ming Chiao Tung University, Hsinchu 300, Taiwan

◊ Department of Electronic Engineering, National Formosa University, Yunlin 632, Taiwan

□ Department of Physics, National Cheng Kung University, Tainan, 70101, Taiwan

& Graduate Institute of Applied Science and Technology, National Taiwan University of Science and Technology, Taipei, 106, Taiwan

∆ Department of Physics, Tamkang University, New Taipei City 25137, Taiwan

♦ Institute of Atomic and Molecular Sciences, Academia Sinica, Taipei 10617, Taiwan

‡ These authors contributed equally.

SUPPLEMENTARY NOTES

1. Synthesis and crystal growth of CIPS sample

CIPS crystals were grown by CVT method using iodine as the transport agent. The growth of high-quality layered CIPS was achieved by preparation of the powdered elements of Cu (99.99% purity), In (99.99% purity), P (99.999% purity), and S (99.999% purity) with stoichiometry (Cu:In:P:S=1:1:2:6) together with an appropriate amount of I_2 (10 mg/cm^3) were put into a quartz ampoule (20 cm in length and 3 cm in inner diameter). Total 10 gram of the powdered mixture with the weight Cu = 1.4686 g, In = 2.6535 g, P = 1.4317 g, and S = 4.4462 g were used. The quartz ampoule with powdered mixture was directly cooled with liquid nitrogen and then sealed in a vacuum environment at ~10^{-6} Torr. Two-step heating process of the layer compound was used for firstly placing two quartz ampoules in a horizontal three-zone furnace at a constant temperature of 600 °C for two days to get synthesized reaction of the starting material, and then setting as 660 °C (heating zone) and 600 °C (growth zone) with a gradient of -2 °C cm^{-1} to the two quartz ampoules for the single-crystal growth. The role of transport agent I_2 is to facilitate the vapor transportation of CIPS from high-temperature end to lower-temperature end for nucleation and growing crystals. The growth reaction kept 360 hours for growing crystals. After the growth, some big and orange-yellow (or brown) like CIPS layered single crystals with area size up to ~ 2-3 cm^2 and a thickness up to 300 μm was obtained. The other batch of small powered-like CIPS crystals was also formed together with the big area crystals. The small crystals have a size area about tens micrometer and a thickness about hundred nanometer. Powdered XRD experiment confirmed that all the as-grown crystals are crystallized in the monoclinic structure of C_c symmetry. The obtained lattice constants of CIPS are a = 6.09 Å, b = 10.56 Å, c = 13.62 Å, and β = 107.1°, respectively.

2. Preparation of V_S-CIPS

V_S-CIPS is prepared in the furnace with a quartz tube and controlled argon atmosphere by a flowmeter and vacuum pump station. The annealing temperature is set at 450 °C and held

for 1 hour. To preserve the ferroelectric polarization, the annealing temperature decreases to 380 ℃ for 10 minutes.

3. Corona poling

A homemade DC needle-plate configured corona poling apparatus is constructed with an array of needles and an aluminum plate. The needle-to-plate gap is set to 2 cm, and the entire setup is leveled via level meters. An external high-voltage source is connected to the needle electrodes while the plate electrode is attached to a grounded microammeter to monitor the received current. The system is confined in a grounded box to minimize atmospheric fluctuations and collect excess ions. During poling, samples are placed beneath the emitting needles to ensure the successful deposition of charged ions. The samples are poled for 30, 45, and 60 minutes with the voltage source (~15 kV) adjusted to maintain the current value at around 80 μA.

4. Material characterizations

The XRD is performed by Bruker D8 Venture X-ray diffractometer (Cu Kα1) with each step of 0.01 degrees and a scan rate of 0.5 s per step. The TEM sample is prepared by exfoliating CIPS bulk crystal into micro-flake on a copper grid. The HRTEM image and SAED pattern are performed in the FEI Tecnai G2-F-20 system. Raman is performed by a homemade system equipped with continuous Nd:YAG 532 nm laser, Olympus microscope (50X objective lens), Andor Kymera 193i-B2 spectrometer, and Andor iDus416 low-noise detector. UV-Vis absorbance spectroscopy is performed by PerkinElmer Lambda 365 UV-Vis spectrophotometer with double-beam design and deuterium and tungsten-halogen light sources. The EPR sample is prepared in powder form, ground from bulk crystal, and characterized by Bruker EMXplus-10/12. The Agilent 7800 ICP-MS is used for vacancy concentration determination.

5. Piezoresponse force microscopy

The PFM sample is exfoliated with tape (SPV-224SRB, Nitto) on the heavily doped silicon substrate. PFM measurements were acquired using a commercial scanning probe

microscope system (Multimode 8, Bruker) with a Nanoscope Controller V. PFM images, off-field hysteresis loops, and cKPFM curves were all carried out under contact-resonance mode with the commercial Pt/Ir-coated tips with spring constant of 2.8 Nm^{-1} (NANOSENSORS PPP-EFM). The tip was driven with an AC voltage amplitude of about 0.5 V and was working at a contact-resonance frequency of about 300 kHz. The off-field hysteresis data and cKPFM curves were obtained via the switching spectroscopic technique with an arbitrary waveform generator (G5100A, Picotest). The temperature-dependent PFM images and quantitative piezoelectric coefficient d_{33} were collected under off-resonance mode by the commercial Pt/Ir-coated tips with spring constant of 7.4 Nm^{-1} (NANOSENSORS PPP-NCSTPt). The thermal application module was used to achieve temperature-dependent measurements with a thermal application controller (TAC, Bruker). During the heating process, an excitation signal of amplitude 1 V working at 7 kHz was applied to capture the temperature-dependent PFM images. A conductive tip was calibrated by a standard sample to extract the quantitative d_{33} values of the sample at individual temperatures.

6. Pump-probe spectroscopy

Time-resolved pump-probe spectroscopy in this study was executed using a dual-color pump-probe system, with a 400 nm (3.1 eV) pump and an 800 nm (1.55 eV) probe. The laser light source was a Ti:sapphire laser with a 5.2 MHz repetition rate, 800 nm wavelength, and 70 fs pulse duration. The pump and probe fluences were set at 77 and 8 μJ cm^{-2}, respectively. The temperature-dependent measurements were carried out in a standard cryostat at ~10^{-3} torr.

7. In situ DRIFT spectroscopy

The *in situ* DRIFT spectra were recorded using a Bruker Tensor 27 FTIR spectrometer with a HgCdTe detector for CO_2 adsorption process and photocatalytic CO_2 reduction reaction.[1, 2] All spectra were acquired with 64 scans with a spectral resolution of 4 cm^{-1}.[3] The acquisition time is ~30 s for each spectrum. A mixture of 4 mg of catalyst and 196 mg of dried KBr powders was used for *in situ* DRIFT measurement. The sample composite was purged with Ar gas for

90 min to remove the residual gases. Subsequently, moist CO_2 gas (CO_2/H_2O) was introduced into the DRIFT cell for 60 min. After CO_2 adsorption process were carried out, the composite was irradiated by using a 100 W Xe lamp for 4 h in a well-sealed cell.

8. In situ XAS measurement

The XAS at Cu K-edge was conducted at BL01C at the Taiwan Light Source (TLS), operating at 1.5 GeV and accommodating a beam current of 360 mA. BL01C, equipped with a Si (111) double-crystal monochromator, delivers an x-ray beam spanning energy from 6 keV to 33 keV. For the in situ XAS measurement, a lab-made magnetic cell was utilized, and the in situ irradiated XAS spectra were acquired using an AM 1.5 solar simulator powered by 500W Xe lamp. The energy resolution for the Cu K-edge was set to approximately 0.4 eV at 8979 eV, with a Cu foil serving as the reference for energy calibration.

9. Photocatalytic CO_2 reduction

The photocatalytic CO_2 reduction is performed via a homemade setup. The reactor is customized and made of aluminum. The reactor equips a quartz window on the top side to allow irradiation with light by a solar simulator (LSH-7320, Class ABA, MiniSol, Newport). CIPS crystal is grounded into powder and weighted for 2 mg for each round of the CO_2 reduction test. The CO_2 gas (99.999%) is set to pass through water before entering the reactor to get a CO_2/H_2O mixture as the reactant. The reactor is purged for at least 30 minutes to remove air and tightly sealed for the reaction. The gastight syringes collect the reaction product to GC-MS (Shimadzu DCMS-QP2020 NX) for analysis. The column used in GC is the Restek CP-PoraBOND Q column. For the isotope experiments, the regular CO_2 gas is replaced with $^{13}CO_2$ (99%, Cambridge Isotope Laboratories, Inc.). A magnetic field is implemented by placing permanent magnets directly beneath the bottom of the reactor. The strength of the magnetic field is measured using a Gauss meter (PCE-MFM 3500-ICA Incl.). Before each round of reaction, the reactor is thoroughly cleaned and baked to prevent contamination.

SUPPLEMENTARY FIGURES

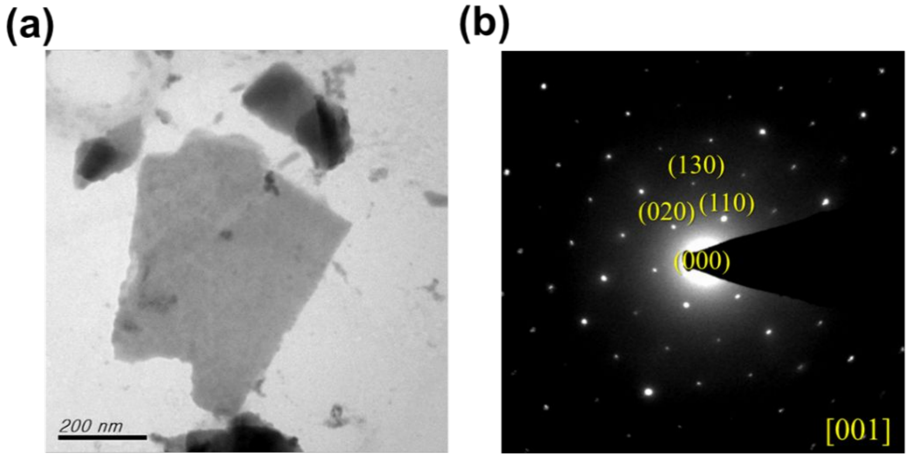

Figure S1. Selected area electron diffraction pattern of the CIPS.

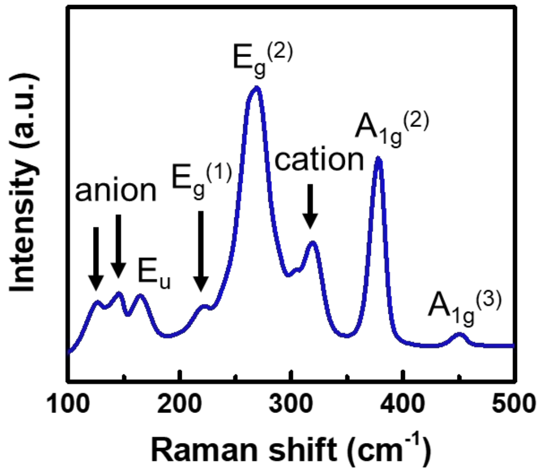

Figure S2. Raman spectrum of the CIPS, which is probed by 532 nm laser.

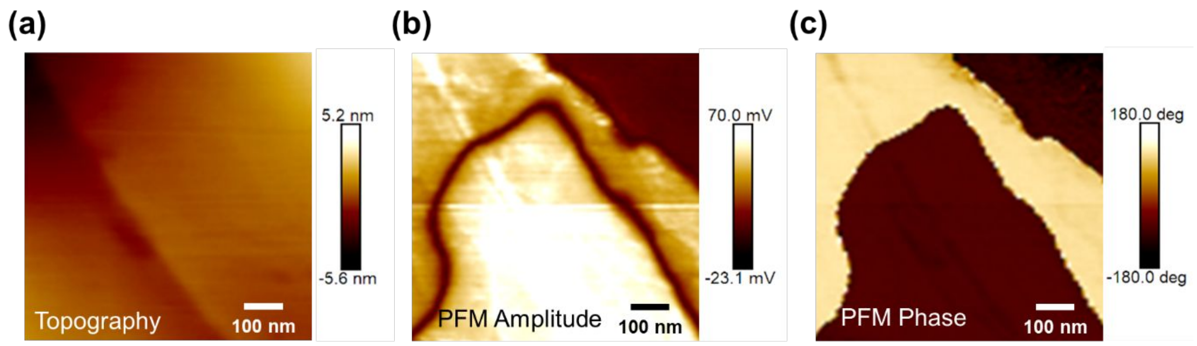

Figure S3. (a) AFM topography image, (b) OP PFM amplitude image, and (c) OP PFM phase image of the CIPS.

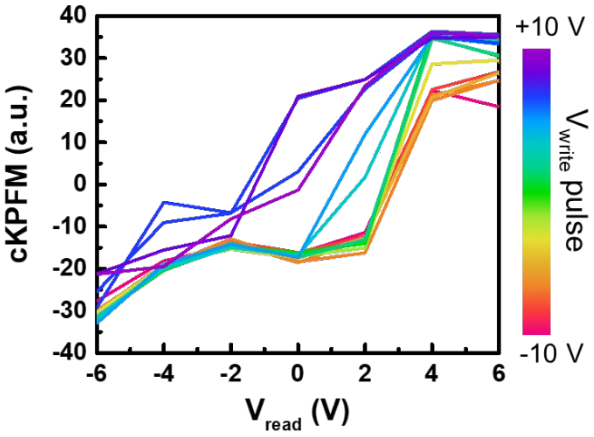

Figure S4. cKPFM curves carried out on the CIPS as a function of V_{read} under a series of V_{write}.

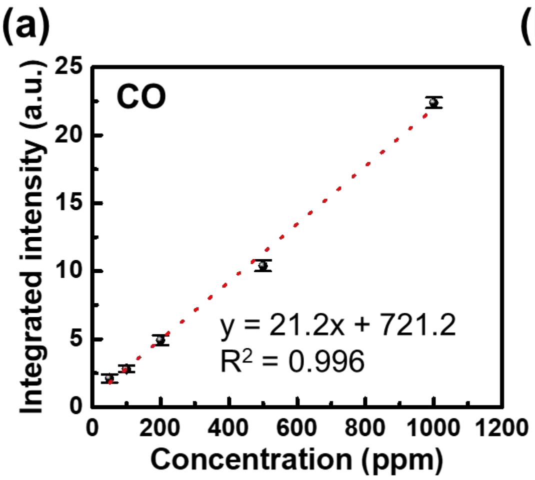

Figure S5. The calibration curves for (a) CO and (b) CH_4 gas concentrations determined by GC-MS.

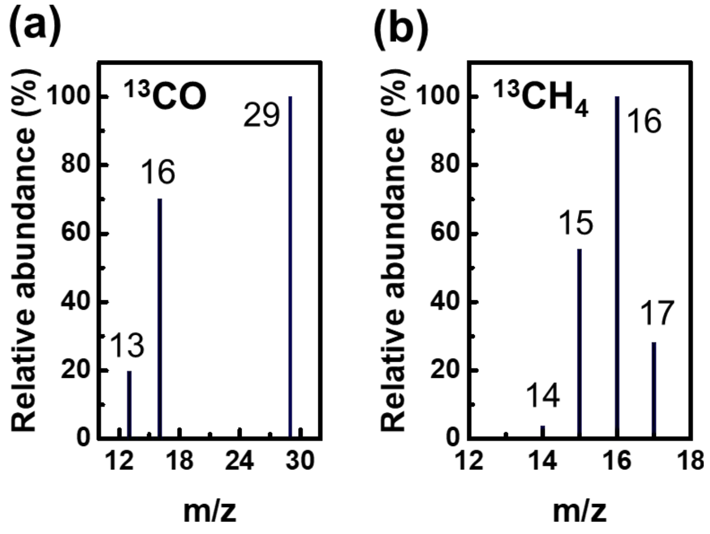

Figure S6. The isotopic mass spectra of (a) ^{13}CO and (b) $^{13}CH_4$ products.

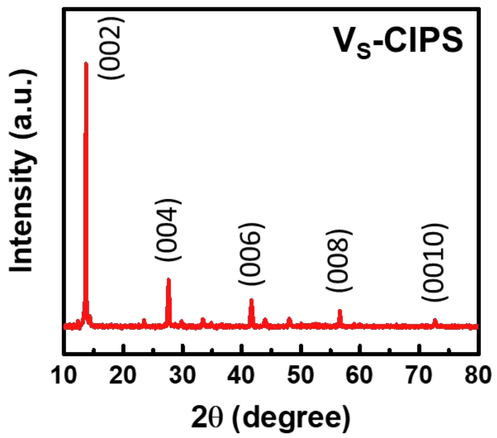

Figure S7. XRD pattern of the V_S-CIPS.

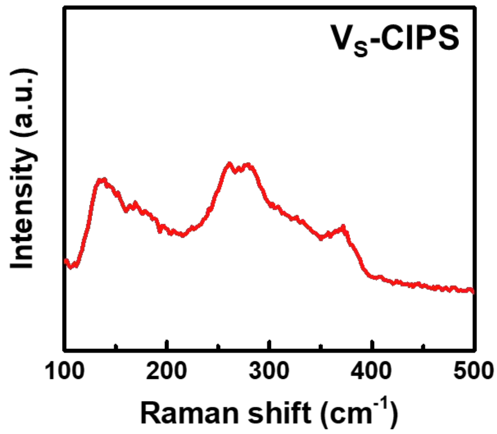

Figure S8. Raman spectrum of the V_S-CIPS.

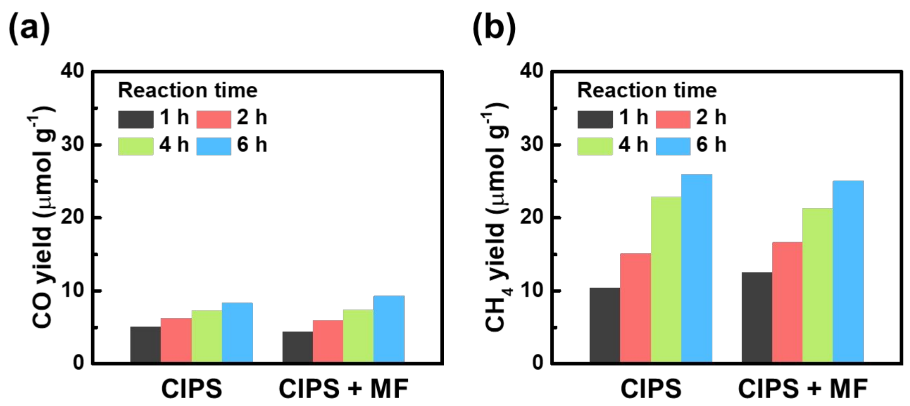

Figure S9. The comparison in CO and CH4 yields of pristine CIPS without and with a magnetic field of 300 mT. (a) CO and (b) CH4 yields after 1, 2, 4, and 6-hour reactions.

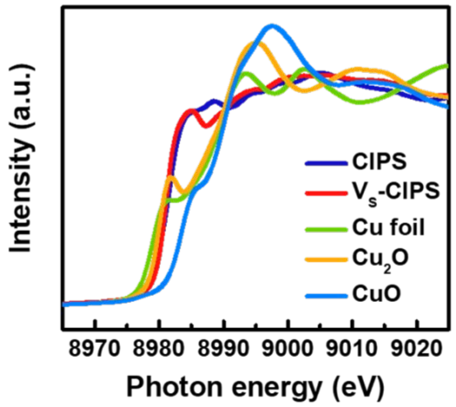

Figure S10. XAS Cu K-edge spectra with various Cu-oxides references (without applying a magnetic field).

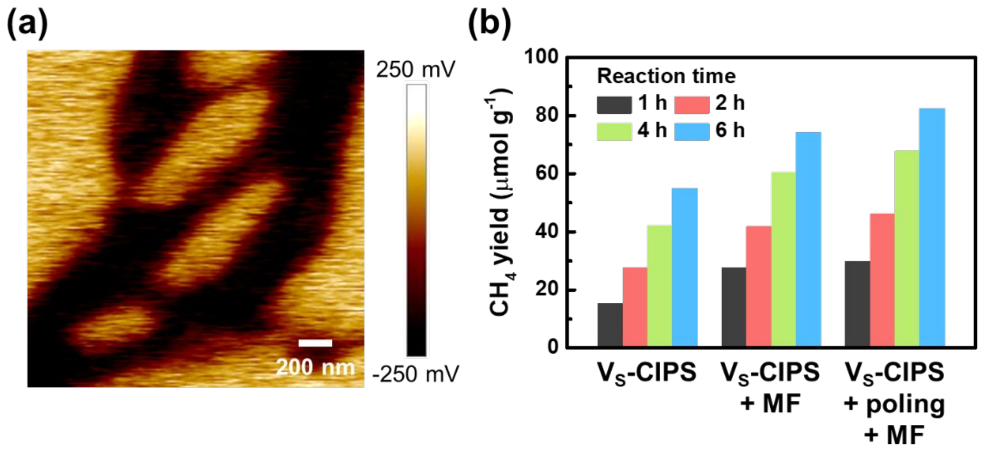

Figure S11. Synergistic manipulation of ferroelectric polarization and spin polarization of V_S-CIPS. (a) OP PFM image and (b) CH_4 yield of of the CIPS after annealing and post-poling processes.

References

(1) Sabbah, A.; Shown, I.; Qorbani, M.; Fu, F.-Y.; Lin, T.-Y.; Wu, H.-L.; Chung, P.-W.; Wu, C.-I.; Santiago, S. R. M.; Shen, J.-L.; Chen, K.-H.; Chen, L.-C. Boosting photocatalytic CO_2 reduction in a $ZnS/ZnIn_2S_4$ heterostructure through strain-induced direct Z-scheme and a mechanistic study of molecular CO_2 interaction thereon. *Nano Energy* **2022**, *93*, 106809.

(2) Kamal Hussien, M.; Sabbah, A.; Qorbani, M.; Hammad Elsayed, M.; Raghunath, P.; Lin, T.-Y.; Quadir, S.; Wang, H.-Y.; Wu, H.-L.; Tzou, D.-L. M.; Lin, M.-C.; Chung, P.-W.; Chou, H.-H.; Chen, L.-C.; Chen, K.-H. Metal-free four-in-one modification of g-C_3N_4 for superior photocatalytic CO_2 reduction and H_2 evolution. *Chem. Eng. J.* **2022**, *430*, 132853.

(3) Chou, T.-C.; Chang, C.-C.; Yu, H.-L.; Yu, W.-Y.; Dong, C.-L.; Velasco-Velez, J.-J.; Chuang, C.-H.; Chen, L.-C.; Lee, J.-F.; Chen, J.-M.; Wu, H.-L. Controlling the oxidation state of the Cu electrode and reaction intermediates for electrochemical CO_2 reduction to ethylene. *J. Am. Chem. Soc.* **2020**, *142*, 2857-2867.
